# Color and Translucency Variation of a One-Shaded Resin-Based Composite after Repeated Heating Cycles and Staining

**DOI:** 10.3390/ma16103793

**Published:** 2023-05-17

**Authors:** Corina Mirela Prodan, Cristina Gasparik, Javier Ruiz-López, Diana Dudea

**Affiliations:** 1 Department of Prosthetic Dentistry and Dental Materials, Iuliu Hatieganu University of Medicine and Pharmacy, 400012 Cluj-Napoca, Romania; corina.prodan@umfcluj.ro (C.M.P.); ddudea@umfcluj.ro (D.D.); 2 Department of Optics, Faculty of Science, Campus de Fuente Nueva, Edificio Mecenas, University of Granada, ibs-Granada, 18071 Granada, Spain; jruizlo@ugr.es

**Keywords:** one-shaded resin-based composite, heating, color difference, color stability

## Abstract

(1) Background: This study aimed to determine the effect of repeated pre-polymerization heating on the color and translucency of a one-shaded resin-based composite and to evaluate whether the heating cycles affect its color stability. (2) Methods: Fifty-six samples of 1-mm thickness were fabricated from Omnichroma (OM) after applying different heating cycles (for one, five, and ten times at 45 °C) before polymerization (*n* = 14/group) and afterwards were stained with a yellow dye solution. CIE L*, a*, b*, C*, h° coordinates were recorded, and color differences, whiteness, and translucency were calculated, before and after staining. (3) Results: Heating cycles significantly influenced the color coordinates, WID_00_, and TP_00_ of OM being higher after one heating cycle and decreasing as the number of heating cycles increased. The color coordinates, WI_D_, and TP_00_ after staining significantly differed for each group. The color and whiteness differences calculated after staining exceeded the acceptability thresholds for all groups. The color and whiteness variations after staining were clinically unacceptable. (4) Conclusions: Repeated pre-polymerization heating induces a clinically acceptable color and translucency change to OM. Although the color changes resulting after staining are clinically unacceptable, increasing the number of heating cycles up to ten times slightly reduces the color differences.

## 1. Introduction

Resin-based composites (RBCs) are widely used as direct restorative materials due to their versatility in different clinical scenarios, good mechanical properties, and excellent aesthetics [1,2,3,4]. Since nanotechnology was introduced in the manufacturing of dental RBCs, nanocomposites have demonstrated superior properties compared to their predecessors [1,5].

Chromatic properties are primarily responsible for the aesthetic integration of a restoration. In most cases, to mimic the natural tooth, the selective reflection of wavelengths is determined by pigments in the composition of the restorative material [4]. More recently, the phenomenon of “structural color” [6] has been used in RBCs to match better the wide color range that characterizes natural dentition. One study [7] reported that spherical nanofillers with a diameter smaller than the wavelength of visible light (<380 nm) could produce structural angle-independent color without adding pigments. Consequently, a “smart chromatic technology” allowed the development of RBCs without pigments, which are claimed to match all VITA shades through reflected wavelengths inside the tooth color space [8,9,10,11]. Omnichroma (OM) is a one-shaded nanofilled RBC that uses structural color and has uniform supra-nanospherical fillers (260 nm spherical particles of SiO_2_-ZrO_2_, 79%wt.) dispersed in a resin matrix containing urethane dimethacrylate (UDMA) and triethylene glycol dimethacrylate (TEGDMA) [8].

Due to the immense advantages of using a one-shaded RBC in different clinical situations, a significant amount of research followed the introduction of OM on the market. The color and optical behavior of OM were intensely investigated in recent studies [4,5,9,12,13,14,15,16]. It was found that the filler system with supra-nanospherical particles demonstrated increased light transmission [17]. Several studies demonstrated that OM has an excellent color adjustment potential (CAP) and can blend with enamel and dentin, leading to perfectly color-matched dental restorations, mostly in cavities surrounded by dental structures [12,14,15,18]. However, one study found that multi-shaded universal RBCs in complex anterior restorations showed better color matching than one-shaded RBCs [13]. A higher thickness of OM would not influence the color adaptation to cavity walls due to its characteristics (high translucency, structural color), as a conventional resin-based composite has a greater color maladjustment to tooth structure as the thickness of the composite increases [19]. In a retrospective study of 2 years, in which OM and another universal resin-based composite were tested for diastema closures and direct veneers, OM reached higher scores for luster and color match [19].

RBCs can be used at room temperature or warmer by heating the syringes or mono-doses in heating units (Calset, ENA Heat, Ease-it) at 54–68 °C [20,21]. Due to a larger delivery system than mono-doses, syringes are preheated several times in daily practice [22]. Preheating RBCs reduces the viscosity and stickiness and improves the handling of materials [23,24,25,26], which leads to an improved marginal adaptation to enamel and dentin, minimizing the risk of secondary caries [27,28]. Moreover, preheating RBCs can reduce the discoloration effect of different staining solutions [29]. However, one study concluded that repeated preheating negatively influenced the flexural strength of RBCs [30]. Considering the temperature level, previous research demonstrated that preheating the composites to 45 °C would not affect the dental pulp [31,32,33], while temperatures over 68 °C may affect the pulp and are not recommended [34].

The Commission Internationale de L’Eclairage (CIE) has developed various color systems, the CIELAB system being the most frequently used in color research in dentistry [35]. The CIELAB color space can be illustrated by a Cartesian system where lightness is represented on a vertical axis by the L* coordinate, and the chromatic coordinates a* and b* are represented on two horizontal axes (red–green and yellow–blue axis, respectively). Based on these coordinates, a difference in color between two objects can be computed using color difference formulae (ΔE_ab_ or ΔE_00_) [35]. The whiteness index (WID) is also calculated from the CIELAB values and expresses the amount of white within a sample [36]. Translucency is a state between transparency and complete opacity and is defined as the color difference between the color coordinates of a sample measured over black and white backgrounds (TP) [37]. Clinical interpretation of color, translucency, and whiteness differences is possible by comparing their values with the respective visual thresholds [35,36,37].

The evolution of the optical properties of RBCs over time is variable [10,38,39,40,41,42]. Color stability is given by the ability of a material to maintain the apparent color after being exposed to challenging conditions such as daylight, humidity, pH modifications, mechanical stress, foods, and beverages with staining potential [10]. Color and translucency stability of RBCs can be influenced by the light-curing process, material aging, and external factors [43,44]. Recent studies concluded that one-shaded RBCs immersed in wine, coffee, and black tea showed more significant color change than multi-shaded RBCs [40,41,42], while nanohybrid and microhybrid resin-based composites showed important color change when stained in turmeric or saffron powder and in grape juice [45].

Although several material-dependent factors were investigated about structural-colored RBCs, to our knowledge, there are no studies investigating the influence of heating cycles on the color and translucency of these materials. Furthermore, the effect of staining in relation to the different number of heating cycles is also unknown. Therefore, the objectives of the study were to assess the effect of repeated pre-polymerization heating cycles on the color and translucency of a one-shaded RBC and to evaluate whether its color stability is affected by the heating cycles. The tested hypotheses were (1) the repeated heating cycles did not affect the color, whiteness, and translucency of the one-shaded RBC, and (2) the staining procedure had the same effect upon the color, whiteness, and translucency of the one-shaded RBC, regardless of the number of heating cycles.

## 2. Materials and Methods

### 2.1. Sample Preparation, Heating, and Staining Protocols

A priori sample size calculation for an effect size f of 0.25, α error probability 0.05, power 0.95, and 4 groups led to a total sample size of 56 (*n* = 14/group). Therefore, fifty-six samples (10.0 mm diameter and 1.0 mm thickness) were fabricated from a novel one-shaded RBC (Omnichroma, Tokuyama Dental, Tokyo, Japan) using different heating cycles.

**Figure 1:**
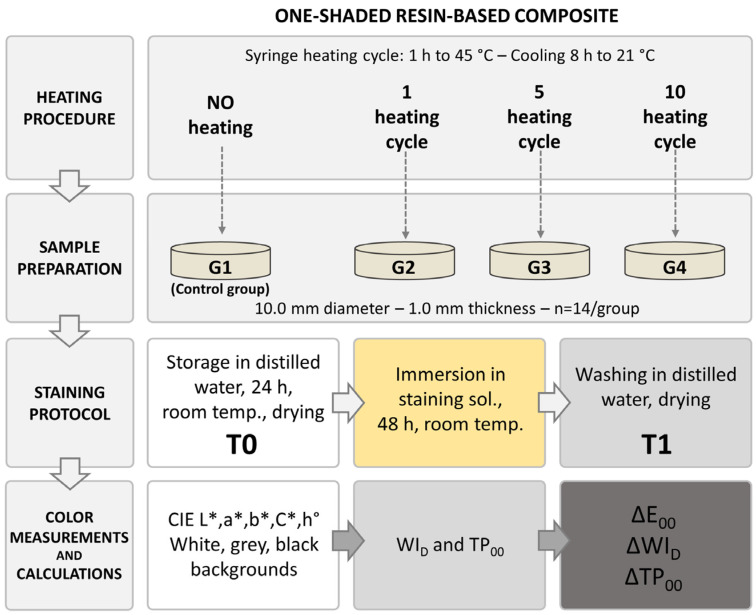
Schematic representation of the experimental methodology: sample fabrication, heating groups, staining protocol, and color measurements.

The sample fabrication is summarized in Figure 1. The first group was considered as the control (group 1), for which the resin-based composite syringe was not heated. For the test groups, the number of heating cycles varied from one (group 2) to five (group 3) or ten times, respectively (group 4). For each group, a different syringe from the same batch was used. The syringes were heated to 45 °C in a resin composite heating unit (Ease-it, Ronvig, Daugaard, Denmark) and were maintained for 1 h in the heating unit to reach the selected temperature. The temperature of the material was verified with a sonde thermometer introduced into the middle of the composite mass. A heating cycle was considered from the syringe’s introduction into the heating unit to the end of the heating time. After each heating cycle, the composite was left for eight hours to cool completely to room temperature (21 °C).

Omnichroma (OM) was packed into a metal cylinder (Porcelain Sampler, Smile Line, Saint Imier, Switzerland), and a Mylar strip was placed over the top of the sample. The samples were polymerized for 40 s on each side using a light-curing unit with an output power of 1800 mW/cm^2^ (Led.H Orto, Woodpecker, Guilin, China) and immersed for 24 h in 3 mL of distilled water, in a dark environment, at room temperature. All specimens were polished with sandpaper (1000 and 2000 grit) for 30 s on each side of the specimen for each granulation and cleaned with distilled water in an ultrasonic bath to remove debris from the surface. The samples were examined for surface defects, and the final thickness (1.00 ± 0.01 mm) was verified using a digital caliper (Z22855, Milomex Ltd., Pulloxhill, UK).

Each specimen was stored for 48 h in 3 mL of staining solution in a dark environment at room temperature. The staining solution was prepared by diluting 2 mL of dye (natural yellow dye, Dr. Oetker, Bielefeld, Germany) in 100 mL distilled water at room temperature (Figure 1). After the staining procedure, the specimens were washed with distilled water and dried.

### 2.2. Color Measurement

The color measurements were performed before (T0) and after (T1) the staining procedure for each heating group (Figure 1). A dental spectrophotometer (SpectroShade Micro, MHT, Niederhasli, Switzerland) was used for recording the CIE L*, a*, b*, C*, h° color coordinates of the samples over white (L* = 91.83; a* = −1.89; b* = 0.16), grey (L* = 46.53; a* = −1.55; b* = −1.28), and black backgrounds (L* = 1.60; a* = 2.09; b* = −2.90). A trained operator performed three consecutive measurements for all samples, and the instrument was calibrated before each measurement.

The total color differences (ΔE_00_) between different groups of heating cycles and for the same groups after staining were calculated using the CIEDE2000 color difference formula [35,46], with the same parameter values used in previous studies [47,48,49]:

(1) $$\Delta E_{00}={\left[{\left(\frac{\Delta L^{\prime }}{k_{L}S_{L}}\right)}^{2}+{\left(\frac{\Delta C^{\prime }}{k_{C}S_{C}}\right)}^{2}+{\left(\frac{\Delta H^{\prime }}{k_{H}S_{H}}\right)}^{2}+R_{T}\left(\frac{\Delta C^{\prime }}{k_{C}S_{C}}\right)\left(\frac{\Delta H^{\prime }}{k_{H}S_{H}}\right)\right]}^{\frac{1}{2}}$$

All values of the color differences were clinically interpreted by comparison with their respective 50:50% visual thresholds for perceptibility (PT_00_) and acceptability (AT_00_), determined in the literature [50,51] and recommended by the Technical Report ISO/TR 28642:2016 [52]: PT_00_ = 0.8 and AT_00_ = 1.8 $\Delta E_{00}$ units. Moreover, the ΔE_00_ units analyzed were divided into the three components: lightness (ΔL_00_), chroma (ΔC_00_), and hue (ΔH_00_), defined as follows [53]:

(2) $$\Delta L_{00}=\frac{\Delta L^{\prime }}{k_{L}S_{L}};\Delta C_{00}=\frac{\Delta C^{\prime }}{k_{C}S_{C}};\Delta H_{00}=\frac{\Delta H^{\prime }}{k_{H}S_{H}}$$

In addition, the whiteness index for dentistry (WI_D_) [48] was calculated for each sample from the measured CIE L* a* b* color coordinates over the grey background using the following formula [36]:

(3) $${\mathrm{WI}}_{D}=0.511L^{*}-2.324a^{*}-1.100b^{*}$$

Whiteness differences (ΔWI_D_) between different groups of heating cycles and for the same groups after staining were calculated [54]. ${\Delta \mathrm{WI}}_{D}$ units were analyzed according to perceptibility (WPT) and acceptability (WAT) 50:50% thresholds for whiteness differences established at 0.72 and 2.62 WI_D_ units, respectively [51,54].

Translucency was evaluated using the translucency parameter (TP) [55], which was calculated as the CIEDE2000 color difference (TP_00_) between the CIE L* a* b* color coordinates of each sample over black (B) and white (W) backgrounds, using the formula [37]:

(4) $${\mathrm{TP}}_{00}={\left[{\left(\frac{{L^{\prime }}_{B}-{L^{\prime }}_{W}}{k_{L}S_{L}}\right)}^{2}+{\left(\frac{{C^{\prime }}_{B}-{C^{\prime }}_{W}}{k_{C}S_{C}}\right)}^{2}+{\left(\frac{{H^{\prime }}_{B}-{H^{\prime }}_{W}}{k_{H}S_{H}}\right)}^{2}+R_{T}\left(\frac{{C^{\prime }}_{B}-{C^{\prime }}_{W}}{k_{C}S_{C}}\right)\left(\frac{{H^{\prime }}_{B}-{H^{\prime }}_{W}}{k_{H}S_{H}}\right)\right]}^{\frac{1}{2}}$$

Differences in translucency (ΔTP_00_) between different groups of heating cycles and for the same groups after staining were evaluated following the 50:50% perceptibility (TPT_00_) and acceptability (TAT_00_) thresholds for translucency: TPT_00_ = 0.62 and TAT_00_ = 2.62 TP_00_ units, respectively [37,51].

### 2.3. Statistical Analysis

The Shapiro–Wilk test was performed to test the normal distribution of the data (α = 0.05). Based on the outcomes of this test, to assess the differences between groups of heating cycles, the Kruskal–Wallis test was used. Contrasts between groups were performed using the Mann–Whitney U test with a Bonferroni correction (p = 0.005). Data were analyzed using the statistical analysis software SPSS Statistics 20.0.0 (IBM Armonk, New York, NY, USA).

## 3. Results

Mean values and standard deviation of CIE L*, a*, b*, C*, h° color coordinates and WI_D_ over grey background, and TP_00_ of the different heating groups before staining (T0), are shown in Table 1.

Heating cycles initially increased the L* coordinate statistically significantly, which stabilized after 5 cycles, since between 5 and 10 heating cycles, no difference was found (*p* = 0.159). The a* coordinate showed no characteristic behavior. It decreased significantly after 1 heating cycle and then significantly increased for 5 heating cycles, while after 10 heating cycles, the value of the a* coordinate was not significantly different from 0 heating cycles (*p* = 0.021). C* and b* color coordinates significantly decreased after 5 heating cycles, but similar to the a* coordinate, after 10 heating cycles, their values were not significantly different from 0 heating cycles (*p* = 0.133 and *p* = 0.101, respectively). The h° coordinate had the opposite behavior compared to the a* coordinate, since it shifted significantly toward the yellow region after 1 heating cycle. However, after 5 heating cycles, the values decreased, while after 10 heating cycles, the values were not significantly different from the control group either (*p* = 0.167).

The WI_D_ values significantly increased after 1 heating cycle, yet after 5 and 10 heating cycles, the WI_D_ values decreased but were still significantly higher than 0 heating cycles. No significant difference was found between 5 and 10 cycles (*p* = 0.458).

TP_00_ significantly decreased after 1 heating cycle; however, after 5 cycles, TP_00_ increased, and after 10 cycles, its values were not significantly different from the control group (*p* = 0.561).

**Figure 2:**
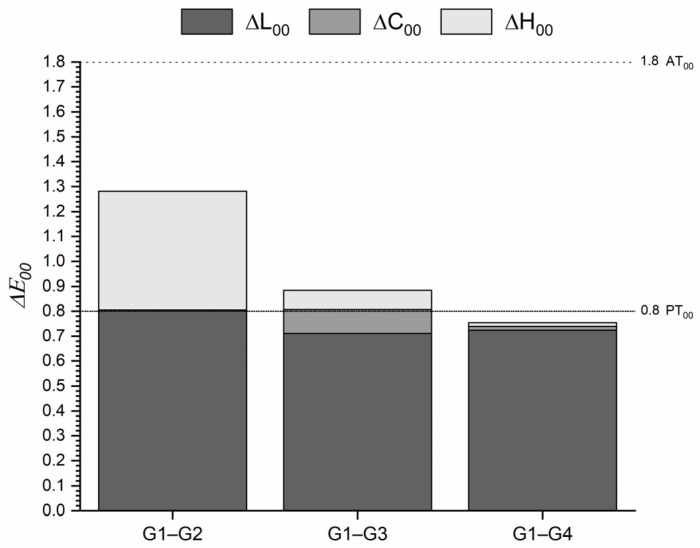
ΔE_00_ of the one-shaded resin-based composite (G1) after 1 (G2), 5 (G3) and 10 (G4) heating cycles. Abbreviations: G1—Group 1; G2—Group 2; G3—Group 3; G4—Group 4.

Figure 2 shows the ΔE_00_ between the tested and the control group for T0. The most important color difference was found between 0 and 1 heating cycles. All comparisons were below the AT_00_, and even the color differences between 0–10 heating cycles were below the PT_00_. For all cases, the lightness shift contributed most significantly to the color difference.

**Figure 3:**
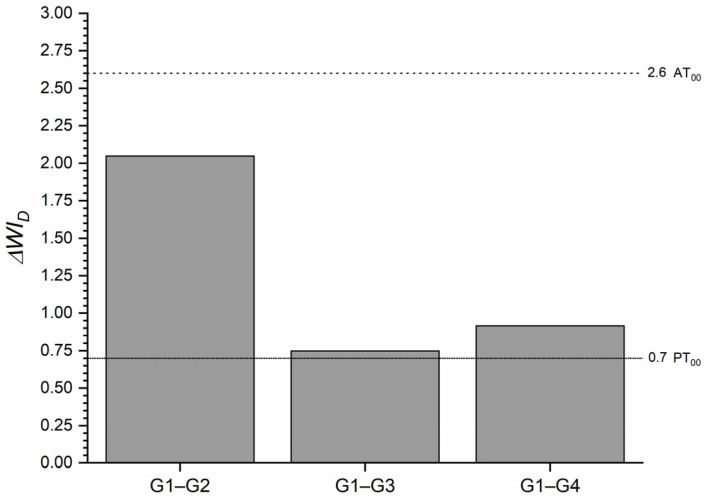
ΔWI_D_ of the one-shaded resin-based composite (G1) after 1 (G2), 5 (G3) and 10 (G4) heating cycles. Abbreviations: G1—Group 1; G2—Group 2; G3—Group 3; G4—Group 4.

The ΔWI_D_ of each tested group and the control group for T0 (Figure 3) was above the WPT in all cases, yet the highest whiteness variation was at 1 heating cycle and the smallest at 5 heating cycles.

**Figure 4:**
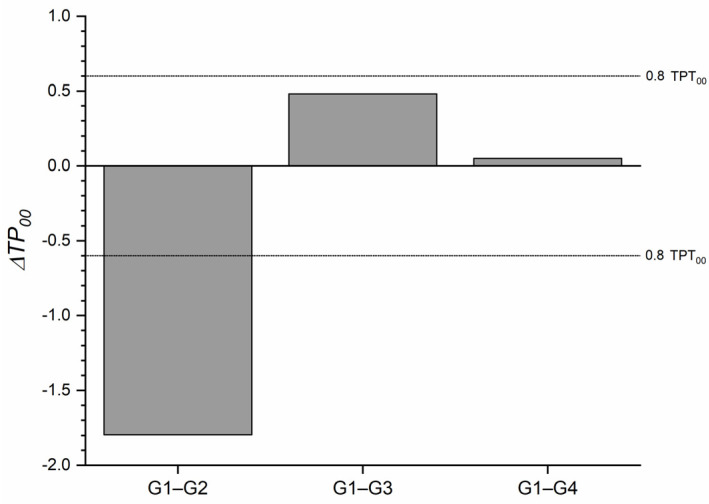
ΔTP_00_ of the one-shaded resin-based composite (G1) after 1 (G2), 5 (G3), and 10 (G4) heating cycles. Abbreviations: G1—Group 1; G2—Group 2; G3—Group 3; G4—Group 4.

The ΔTP_00_ between all tested groups and the control groups for T0 is shown in Figure 4, where only at 1 heating cycle the ΔTP_00_ value exceeded the TPT_00_. However, after 5 heating cycles, the ΔTP_00_ increased without becoming perceptible.

The results of the experimental staining of OM subjected to different regimens of heating before polymerization are presented in Table 2, where the mean values and standard deviations of L*, a*, b*, C*, h° color coordinates and WI_D_ over grey background and TP00 are shown.

After the staining process, all the color coordinates, WI_D_, and TP_00_ of the control and tested groups were statistically significantly different from their respective baseline values (*p* < 0.005). The L* and a* coordinates slightly decreased after staining, while b*, C*, and h° coordinates significantly increased. The WI_D_ of the stained samples was lower than the baseline, while the TP_00_ increased.

**Figure 5:**
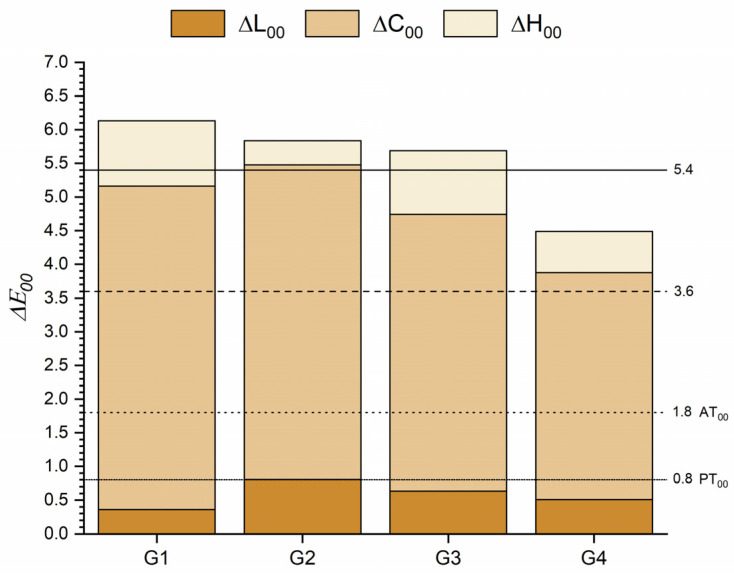
ΔE_00_ after staining of the one-shaded resin-based composite for each group of heating cycles. (ΔE_00_ between T0 and T1). Abbreviations: G1—Group 1; G2—Group 2; G3—Group 3; G4—Group 4; T0—Before staining procedure; T1—After staining procedure.

Figure 5 shows the color differences after the staining procedure, where it can be observed that for all groups, the ΔE_00_ exceeded the AT_00_. For groups with 0, 1, and 5 heating cycles, the color difference values were more than three times higher than the AT_00_, while for the group with 10 heating cycles, the values were more than two times higher. In all situations, ΔC_00_ had the most significant contribution to the total color difference.

**Figure 6:**
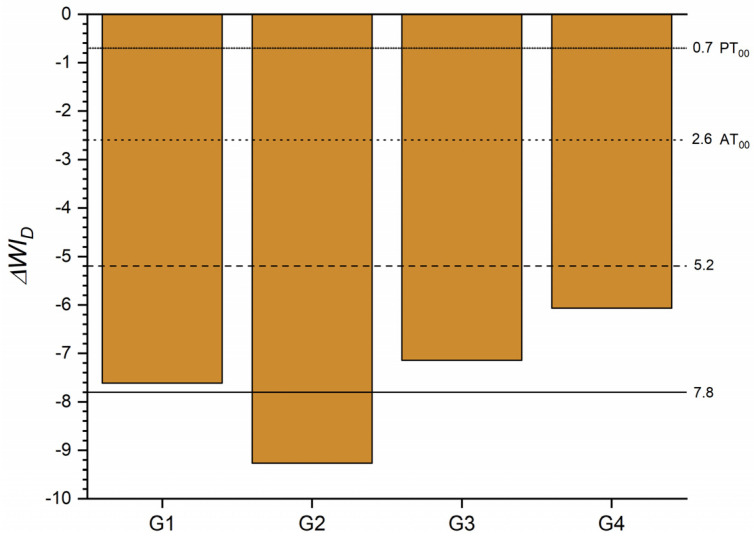
ΔWI_D_ after staining of the one-shaded resin-based composite for each group of heating cycles. (ΔWI_D_ between T0 and T1). Abbreviations: G1—Group 1; G2—Group 2; G3—Group 3; G4—Group 4; T0—Before staining procedure; T1—After staining procedure.

The ΔWI_D_ values after the staining procedure are shown in Figure 6, decreasing below twice the value of WAT for all groups, being even three times lower for the group with one heating cycle.

**Figure 7:**
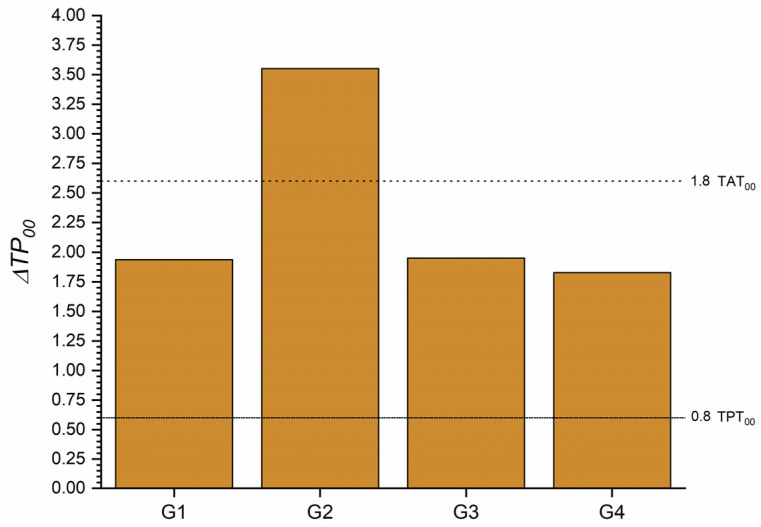
ΔTP_00_ after staining of the one-shaded resin-based composite for each group of heating cycles. (ΔTP_00_ between T0 and T1). Abbreviations: G1—Group 1; G2—Group 2; G3—Group 3; G4—Group 4; T0—Before staining procedure; T1—After staining procedure.

The ΔTP_00_ values obtained after the staining procedure are shown in Figure 7 and exceeded the TPT_00_ in all situations with similar values, except for group 2, which was higher than the TAT_00_.

## 4. Discussion

Heating RBCs before curing is a clinical procedure frequently used by dentists to improve materials handling before placement in the oral cavity.

Even though preheating of the resin composite is not mandatory, the advantages of this procedure have been proven from a clinical point of view [22]. In addition, preheating of the resin-based composites is indicated by some manufacturers. The heating effect was previously investigated but most studies focused on the effect of one heating cycle [29,56,57]. However, it is common in clinical practice to reuse composite syringes, and therefore, heating of the same syringe can occur up to 20 times, mainly if a multi-layering technique is used [30].

Our results showed that all color coordinates, as well as WI_D_ and TP_00_, varied after repeated heating cycles; therefore, the first tested hypothesis was rejected. The most significant variation was observed for lightness, which increased after 1 heating cycle but remained relatively constant after 5 or 10 heating cycles. However, a*, b*, C*, h° coordinates, and TP_00_ after 10 heating cycles, were not significantly different from the unheated control group. Although the WI_D_ increased significantly after one cycle, it dropped slightly after 5 cycles but remained higher than the reference WI_D_ values corresponding to the unheated control group. Moreover, the TP_00_ values calculated for 1.0-mm thick samples fabricated from OM without heating were similar to those reported by a previous study that analyzed the optical behavior of different one-shaded RBCs [9].

Although the color coordinates varied significantly after repeated heating cycles, the color differences were below the acceptability threshold even after 10 heating cycles (Figure 2). In particular, the color, whiteness, and translucency differences found were higher after one heating cycle and followed a decreasing trend as the number of heating cycles increased. This finding has an important clinical relevance since the results of our study demonstrated that repeated heating of OM induces a clinically acceptable color change.

Previous research investigated the effect of preheating on the color stability of a nanohybrid composite [29]. In that study, the RBC was heated once to 68 °C, and after light-curing and surface finishing, the samples were immersed in distilled water, coffee, and tea. The authors concluded that the preheated RBC showed significantly lower discoloration than the unheated group when immersed in coffee, but the difference was not significantly different for the immersion in tea [29].

Translucency changes in an RBC can occur due to light-curing or aging [43]. In our study, the TP_00_ dropped significantly after one heating cycle, but after 5 and 10 heating cycles, the values were close to those of the no-heating group (Figure 4). This result could be explained by the increase in the L* coordinate of the RBC. Possibly after the first heating cycle, the homogeneity of the material was affected, but after repeated heating, it returned to the baseline. Nevertheless, additional studies are necessary to support this speculation.

Different studies investigating OM have aimed to characterize it by evaluating mechanical properties, light transmission, and cell toxicity. The filler system with supra-nanospherical particles demonstrated an increased light transmission [17]. Furthermore, the cell viability was comparable for structural and pigment-colored materials [4]. However, it was concluded that the particular composition of structural-colored materials induced similar or poorer mechanical properties than the pigment-colored materials [16], which is associated with higher sensitivity to aging and lower reliability [17].

Currently, there is no agreement on how heating influences some of these properties. Previous research showed that after heating (one cycle of 40 s), the mechanical properties of RBC were unaltered [27], while another study demonstrated that after 40 preheating cycles of 12 min to 45 °C, the mean flexural strength of both microhybrid and nanofilled RBC showed a significant decrease [30]. Furthermore, another study concluded that when a universal RBC and a silorane composite were preheated for 40 cycles of 12 min at 55–60 °C, the color changes were more significant than for the unheated composites [38].

The second tested hypothesis was also rejected since the color coordinates, WI_D_, and TP_00_ of the samples after the staining process were significantly different for each heating group. Furthermore, the number of heating cycles affected all the parameters analyzed since the ΔE_00_, ΔWI_D,_ and ΔTP_00_ values obtained decreased sequentially from 1 to 10 heating cycles. However, the values were above the acceptability thresholds in all the studied parameters except ΔTP_00_.

The ΔE_00_ values obtained for all heating groups were greater than three times the AT_00_ after staining, which, according to the AT_00_ rating from Paravina et al. [51], would represent an extremely unacceptable match (Figure 5), except for group 4, which after 10 heating cycles showed a clearly unacceptable match [51], being only more than twice the AT_00_. In all cases, ΔE_00_ were mainly due to the increase in chroma, followed by the shift toward the yellow region of the color space. These results are consistent due to the yellow die used to prepare the staining solution, which was selected because it is often found in many foods and beverages.

Consequently, the WI_D_ values decreased for all the heating groups analyzed with a similar pattern, obtaining a ΔWI_D_ with a clearly unacceptable match, according to the WAT_00_ rating described by Paravina et al. [51], except for group 2, which, after a single heating cycle, reached an extremely unacceptable match (Figure 6). These results are consistent with the color variations obtained and the staining procedure, where again, it is notable to find the slightest variations in group 4, after 10 heating cycles.

One study concluded that the color changes of a universal RBC and a silorane-based composite after immersion in tea were lower for the heated group than the unheated group [38]. In our study, the color changes resulting after staining the one-shaded RBC were similar for the unheated group and heating groups 2 and 3 (after 1 and 5 heating cycles). After 10 heating cycles, the color change after the staining procedure was significantly lower than for the unheated group, which is consistent with the results obtained by Abed Kahnamouei et al. [38], although their results were obtained after 40 heating cycles.

It is well known that increasing the polymerization temperature leads to a higher degree of dimethacrylate monomers conversion [58]. This effect is limited to near 90 °C for bisphenol A-glycidyl methacrylate (Bis-GMA) and ethoxylated bisphenol-A dimethacrylate (Bis-EMA). Above this temperature, the degree of conversion drops due to reactant evaporation and photoinitiator degradation [58,59]. However, although the degree of monomer conversion influences the chemical properties of the resin composites, one study [20] concluded that preheating a nanohybrid composite to 60 °C increased the monomer conversion but did not influence the optical properties significantly (color stability and opacity variation).

Although there are recent studies published on the color stability of OM, the results reported are inconsistent. When OM was compared to a multi-shaded nanocomposite to evaluate the color stability and gloss retention when immersing them in tea and red wine, it was concluded that for OM, the ΔE_00_ values were statistically significantly higher [20]. Similar results were reported by another study investigating the color stability of two one-shaded RBCs compared to multi-shaded RBCs [42]. These results could be explained by the chemical composition of the organic matrix of the one-shaded RBCs. Both OM and Vitra Unique, another one-shaded RBC, have in their composition TEGDMA, which might be responsible for the higher susceptibility to discoloration due to its higher water absorption properties than Bis-GMA [60].

On the contrary, another study reported that when OM was compared with other nanofilled resin composites, no significant differences were found regarding color stability after immersion in tea [41]. Moreover, other research concluded that accelerated aging effects were material-dependent, where OM exhibited significantly lower color change than other tested RBCs in general [10].

The current study used different RBC syringes of the same material (OM) for sample fabrication. However, this material is a one-shaded RBC without an inherent color and with excellent color adjustment potential. In addition, RBC syringes from the same lot and with the same expiry date were used. On the other hand, although only one type of staining solution was evaluated, it induced a significant color change in the one-shaded RBC samples.

Another limitation of the study is that the initial roughness of the samples was not evaluated using surface roughness measurement methods. However, the polishing of the samples was standardized and the variation of the surface roughness within and between the groups was controlled.

Further studies should be carried out, including other one-shaded RBCs, different staining solutions, and artificial aging to assess the color stability and to compare their optical behavior upon repeated heating. Moreover, further studies on the variation of the color adjustment potential of one-shaded RBCs after repeated heating and staining would be of high clinical interest.

## 5. Conclusions

Within the limitations of the present study, it was concluded that, in general, the color, whiteness, and translucency of OM showed the highest variations after the first heating cycle. Although clinically acceptable, these changes decreased as heating cycles increased. Except for the translucency changes, the color and whiteness variations that occurred after the staining were clinically unacceptable; however, these changes were smaller as the number of heating cycles increased.

## Figures and Tables

**Table 1:** Mean values and standard deviation of color coordinates, WI_D_ (over grey background) and TP_00_ before staining (T0).

	L*	a*	b*	C*	h°	WI_D_	TP_00_
Group 1 (0 cycles)	74.0 (1.1)	1.2 (0.2) ^a^	14.0 (0.8) ^a,b^	14.1 (0.8) ^a,b^	84.9 (1.1) ^a^	19.6 (0.7)	23.9 (0.9) ^a^
Group 2 (1 cycle)	75.4 (0.4)	0.6 (0.2)	14.1 (0.7) ^a,c^	14.2 (0.7) ^a,c^	87.5 (0.8)	21.6 (0.5)	22.1 (0.5)
Group 3 (5 cycles)	75.1 (1.0) ^a^	1.4 (0.2)	13.5 (0.6)	13.6 (0.6)	84.1 (1.0)	20.3 (0.6) ^a^	24.4 (0.3) ^b^
Group 4 (10 cycles)	75.1 (1.0) ^a^	1.1 (0.2) ^a^	13.8 (0.7) ^b,c^	13.9 (0.7) ^b,c^	85.3 (0.9) ^a^	20.5 (0.6) ^a^	24.0 (0.7) ^a,b^

Same lowercase letter, for each column, shows no statistically significant difference among the different groups evaluated (*p* > 0.005).

**Table 2:** Mean values and standard deviation of color coordinates, WI_D_ (over grey background) and TP_00_ after the staining procedure (T1).

	L*	a*	b*	C*	h°	WI_D_	TP_00_
Group 1 (0 cycles)	72.1 (0.7)	−0.7 (0.5)	24.2 (1.7)	24.2 (1.7)	91.7 (1.0)	11.9 (1.0)	25.9 (0.7)
Group 2 (1 cycle)	72.5 (0.5)	−0.6 (0.4)	23.8 (1.5)	23.8 (1.5)	91.4 (0.8)	12.3 (1.0)	25.7 (0.5)
Group 3 (5 cycles)	72.6 (0.3)	−0.3 (0.3)	22.4 (1.1)	22.4 (1.1)	90.7 (0.7)	13.2 (0.6)	26.4 (0.3)
Group 4 (10 cycles)	73.0 (0.5)	0.0 (0.4)	20.9 (1.1)	20.9 (1.1)	90.0 (1.0)	14.4 (0.7)	25.8 (0.5)

All comparisons between groups for each evaluated parameter were statistically significant (*p* < 0.005).

## Data Availability

The data presented in this study are available on request from the corresponding author.
